# The Effects of Transcranial Direct Current Stimulation on the Cognitive and Behavioral Changes After Electrode Implantation Surgery in Rats

**DOI:** 10.3389/fpsyt.2019.00291

**Published:** 2019-05-07

**Authors:** Jooyoung Oh, Jinsil Ham, Dongrae Cho, Jin Young Park, Jae-Jin Kim, Boreom Lee

**Affiliations:** ^1^Department of Psychiatry, Gangnam Severance Hospital, Yonsei University Health System, Seoul, South Korea; ^2^Institute of Behavioral Science in Medicine, Yonsei University College of Medicine, Seoul, South Korea; ^3^Department of Biomedical Science and Engineering (BMSE), Institute of Integrated Technology (IIT), Gwangju Institute of Science and Technology (GIST), Gwangju, South Korea

**Keywords:** brain stimulation, transcranial direct current stimulation, delirium, rat, electrophysiology, connectivity

## Abstract

Postoperative delirium can lead to increased morbidity and mortality, and may even be a potentially life-threatening clinical syndrome. However, the neural mechanism underlying this condition has not been fully understood and there is little knowledge regarding potential preventive strategies. To date, investigation of transcranial direct current stimulation (tDCS) for the relief of symptoms caused by neuropsychiatric disorders and the enhancement of cognitive performance has led to promising results. In this study, we demonstrated that tDCS has a possible effect on the fast recovery from delirium in rats after microelectrode implant surgery, as demonstrated by postoperative behavior and neurophysiology compared with sham stimulation. This is the first study to describe the possible effects of tDCS for the fast recovery from delirium based on the study of both electroencephalography and behavioral changes. Postoperative rats showed decreased attention, which is the core symptom of delirium. However, anodal tDCS over the right frontal area immediately after surgery exhibited positive effects on acute attentional deficit. It was found that relative power of theta was lower in the tDCS group than in the sham group after surgery, suggesting that the decrease might be the underlying reason for the positive effects of tDCS. Connectivity analysis revealed that tDCS could modulate effective connectivity and synchronization of brain activity among different brain areas, including the frontal cortex, parietal cortex, and thalamus. It was concluded that anodal tDCS on the right frontal regions may have the potential to help patients recover quickly from delirium.

## Introduction

Delirium is described as an acute, transient, fluctuating disturbance in attention, consciousness, and cognition (1). Delirium is a common condition in general hospitals (2), especially in the intensive care unit (3), and is a potentially life-threatening clinical syndrome (4). Delirium can frequently occur after surgery (5, 6), which can lead to increased morbidity, mortality, functional impairment, cognitive dysfunction, and increased medical costs (7, 8).

Previous studies have suggested that delirium is related to functional brain abnormalities (9, 10). Considering that proper cognitive function requires connectivity among brain regions (11), and that patients with delirium have cognitive problems, we would expect that delirium is related to disturbances in functional brain networks. Studies using electroencephalography (EEG) have shown that delirium is characterized by excessive EEG slowing, demonstrated by increases in theta and delta power (10). This EEG change may be resolved after an episode of delirium (12). In terms of neuroimaging studies, our group has previously reported that disruptions in reciprocity between the dorsolateral prefrontal cortex and posterior cingulate cortex, as well as the reduction of functional connectivity among subcortical regions, may contribute to the pathophysiology of delirium (9).

Generally, human EEG signals are measured in a noninvasive manner, whereas animal EEG can be easily measured invasively, creating a high signal-to-noise ratio (13). In this aspect, animal studies can be helpful in better understanding the brain mechanisms underlying delirium through these high signal-to-noise ratios. However, there are few studies on animal models for delirium. In one such study, systemic inflammation was induced using lipopolysaccharide (14), consequentially causing acute cognitive deficits, relevant to aspects of delirium. Anticholinergic drugs have also been utilized to induce delirium-like symptoms in rats (15–18) and have been reported to lead to delirium-like EEG changes, including EEG slowing. In addition, a simple laparotomy under isoflurane anesthesia has been used to produce animal models of postoperative delirium (19, 20). Considering that delirium has many heterogeneous contributing factors (21), previous studies that induce delirium in a variety of ways may be justified; however, the evaluation of delirium should be performed through the assessment of behavioral changes, as clinical diagnoses of delirium are generally made based on the cognitive and behavioral manifestations including both attentional deficits and fluctuating course (2). It should be noted that fluctuating course is an essential feature of delirium, especially for the differential diagnosis with dementia, and may be due to the multiple fluctuating changes of the contributing factors such as systemic inflammation, cerebral metabolism, drugs, autonomic nervous system, and neurotransmitters (2, 22). However, some previous studies on animal models of delirium did not assess fluctuating course (14, 15). In this context, a method that measures behavior at multiple times to prove fluctuation has been well presented in previous studies (19, 20); however, besides the behavioral assessments, there was lack of supporting evidence to prove delirium in other measures such as functional brain changes. Although they reported elevations in the levels of α-synuclein and S100β in their model, cellular/molecular biomarkers of delirium have not been sufficiently investigated to be reliable (23) compared to functional brain changes examined using EEG (12, 24–27) and fMRI (9, 28, 29). Taken together, simultaneous serial assessments of both behavioral changes and functional brain changes are needed to more clearly determine whether an animal experiences an episode of delirium or not.

While there is strong evidence for altered neural activity and connectivity in patients with delirium, the prevention and management of delirium remains elusive. Generally, once delirium occurs, we treat the cause of delirium and provide environmental and supportive interventions as nonpharmacological treatments (30, 31); however, antipsychotic medications such as haloperidol are also usually considered for the treatment of delirium (32). Despite the use of medications, they are unable to significantly alter the duration of delirium (33). Furthermore, preventive methods have not been sufficiently investigated compared to post-treatment methods, and the studies that have compared these interventions have shown negative results (34, 35). Transcranial direct current stimulation (tDCS) offers a modality that can modulate brain activity noninvasively using weak currents applied through scalp electrodes (36). Many studies have shown that anodal tDCS can enhance cognitive functions, including attention deficits, which comprise a core symptom of delirium (14, 37). In particular, application of tDCS on the right frontal regions appears to be associated with increased attention, recognition of surrounding objects, and self-monitoring (38, 39), suggesting that it may have the potential to prevent, or quickly recover, symptoms of delirium. To date, the effects of tDCS on delirium have not been studied in humans or animal models.

Accordingly, the aim of this study was to investigate the potential effects of tDCS on delirium including cognitive and behavioral changes after surgery. We expected that postoperative delirium could be induced by performing microelectrode implantation surgery under isoflurane anesthesia similar to the previous studies that performed laparotomy (19, 20). Furthermore, electrophysiological signals were measured and analyzed, which allowed us to evaluate the mechanisms underlying postoperative delirium and the effects of tDCS at the neural signal level. Several behavioral tests were also performed to investigate the symptoms of delirium, including attentional deficit and fluctuating course. In particular, the animals were serially evaluated at 6, 9, 24, and 48 h, which is the most frequently evaluated work in terms of behavioral measurements in animal models of delirium. This allowed us to further detect the fluctuating course of behaviors. Furthermore, measuring EEG signals in rats allows us to obtain signals from deep brain regions including the thalamus and thus to further elucidate the neural mechanisms of delirium.

## Materials and Methods

### Animal Care and Use

**Figure 1:**
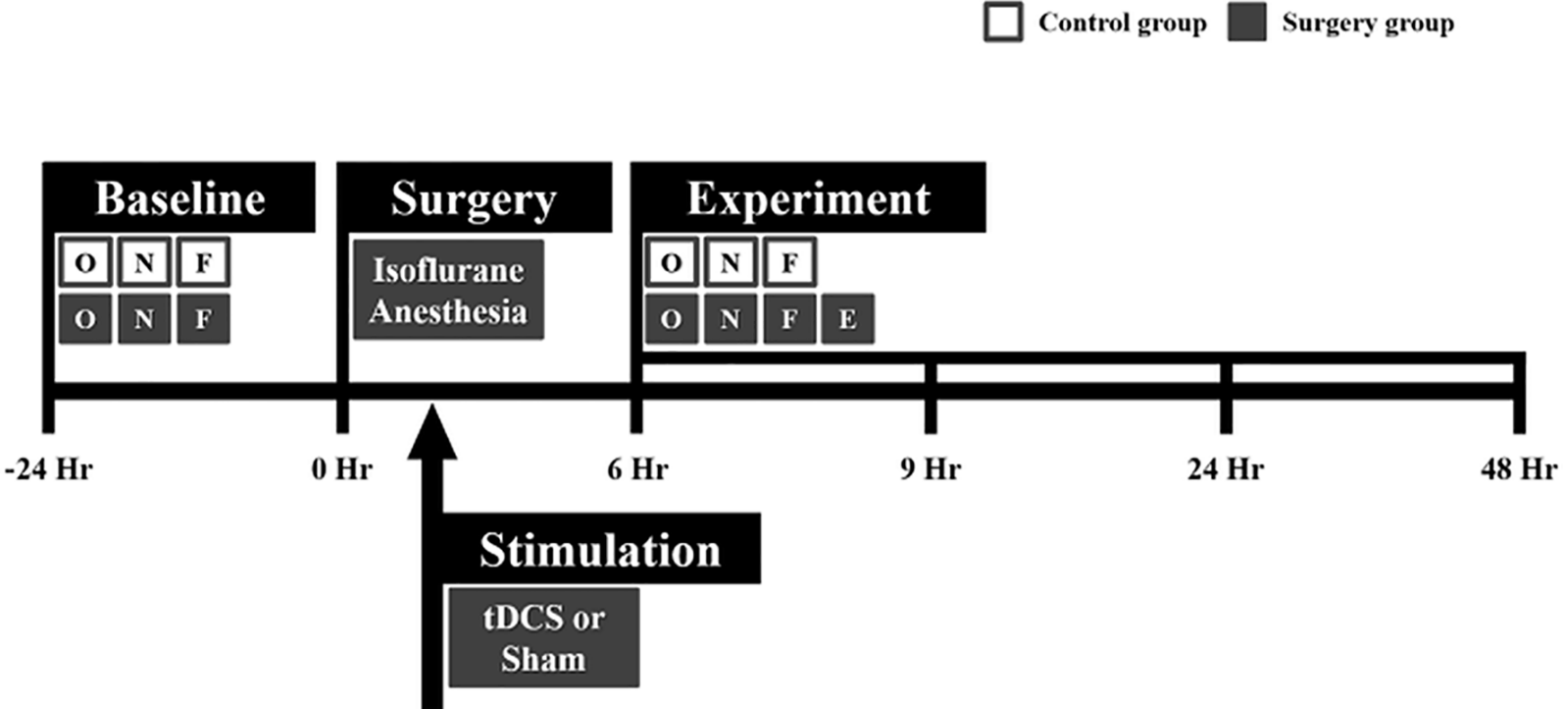
Flowchart of the whole experiment. Microelectrode implantation surgery was performed under isoflurane anesthesia. Immediately after surgery, transcranial direct current stimulation (tDCS) or sham stimulation was applied to the right frontal area for 20 min. All rats in the control and surgery groups underwent behavioral tests at every time point (before and after surgery). Local field potential (LFP) data were only acquired in the surgery group 6, 9, 24, and 48 h after surgery. The behavioral tests and LFP recording were sequentially performed in the order indicated in the flowchart. The white and gray boxes indicate the control and surgery groups, respectively. O = open field test, N = novel object recognition test, F = buried food test, and E = EEG measurement.

All animal experiments complied with the guidelines of the Institutional Animal Care and Use Committee (IACUC) of the Gwangju Institute of Science and Technology (GIST). This study was approved by the institutional review board at GIST. The experiment was designed as shown in **Figure 1**. In this study, 26 male Sprague–Dawley rats weighing 421.98 ± 84.63 g (mean ± standard deviation) were used. The rats were single-caged in a controlled animal facility at a constant temperature (21 ± 1°C). The animals were 15.92 ± 3.89 weeks old at the time of the experiment. The rats were maintained in a room with a 12-h light–dark cycle. They were randomly assigned to the tDCS group (n = 10), the sham group (n = 10), or the control group (n = 6).

### Microelectrode Implantation Surgery: Rat Model of Postoperative Delirium

**Figure 2:**
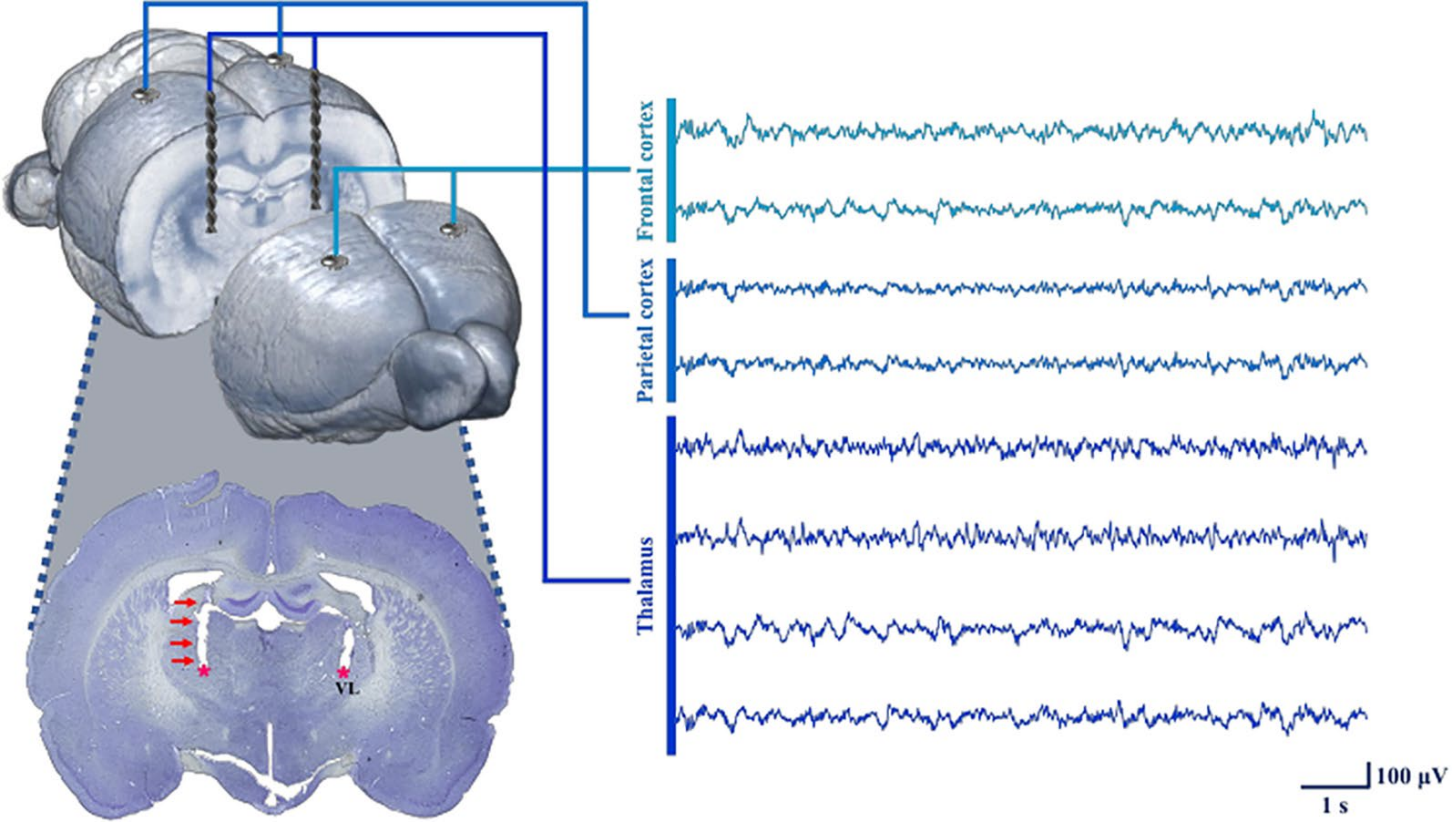
Process used for neural signal acquisition at each target point using the inserted electrodes. To measure LFPs, microelectrodes were implanted into the frontal and parietal cortices. In four animals (two from tDCS group, two from sham group), depth electrodes were also inserted in the thalamus. The depth electrodes successfully targeted the VL (ventrolateral nucleus of the thalamus), as shown in the histology image. All of the neural signals were reliably obtained under our recording conditions.

Rats in the tDCS and sham groups underwent neurosurgery under isoflurane anesthesia to induce postoperative delirium. We expected electrode-implant neurosurgery under anesthesia to induce postoperative delirium, similar to simple laparotomy performed under isoflurane anesthesia (19, 20). The microelectrodes were implanted in the frontal lobe, parietal lobe, and thalamic region to measure local field potentials (LFPs). The rats were anesthetized using isoflurane in 100% oxygen during the entire surgery procedure. Five percent isoflurane was used for 15 min during the induction period in a transparent acrylic chamber, and 1.5–3% isoflurane was utilized to maintain the anesthesia during surgery *via* a mask. The head was fixed inside a stereotaxic instrument. The incisor bar was adjusted to ensure that the heights of bregma and lambda were equal. Microelectrodes were implanted through small holes after a durotomy, and the following brain regions were selected as regions of interest for measuring LFPs: left frontal lobe, right frontal lobe, left parietal lobe, and right parietal lobe. Four rats (two from the tDCS group and two from the sham group) were further implanted with depth electrodes to acquire LFPs from the left thalamus and the right thalamus. The stereotaxic coordinates for the targeted areas (in mm) were as follows (anterior or posterior to bregma, midline to lateral, surface to depth): left frontal lobe (+5.5, +3, 0), right frontal lobe (+5.5, −3, 0), left parietal lobe (−4.36, +4, 0), right parietal lobe (−4.36, −4, 0), left thalamus (−2.4, +2.5, −6.15), and right thalamus (−2.4, −2.5, −6.15). For tDCS stimulation, we also placed an epicranial electrode holder on the right frontal scalp (+2, −1.5, 0). **Figure 2** illustrates the process used to obtain the signal, as well as a histology image showing that the depth electrodes were appropriately located in the thalamus. The electrodes and connectors were tightly fixed using bone cement. After the surgery, an analgesic (ketoprofen, 1.5 mg/kg, Uni Biotech; Chungnam, Korea) and an antibiotic (ceftezole, 1.5 mg/kg, Shin Poong Pharm Co.; Seoul, Korea) were injected intramuscularly once a day for 3 days. The total operation time including the anesthesia induction time was about 2 h on average.

### Transcranial Direct Current Stimulation

**Figure 3:**
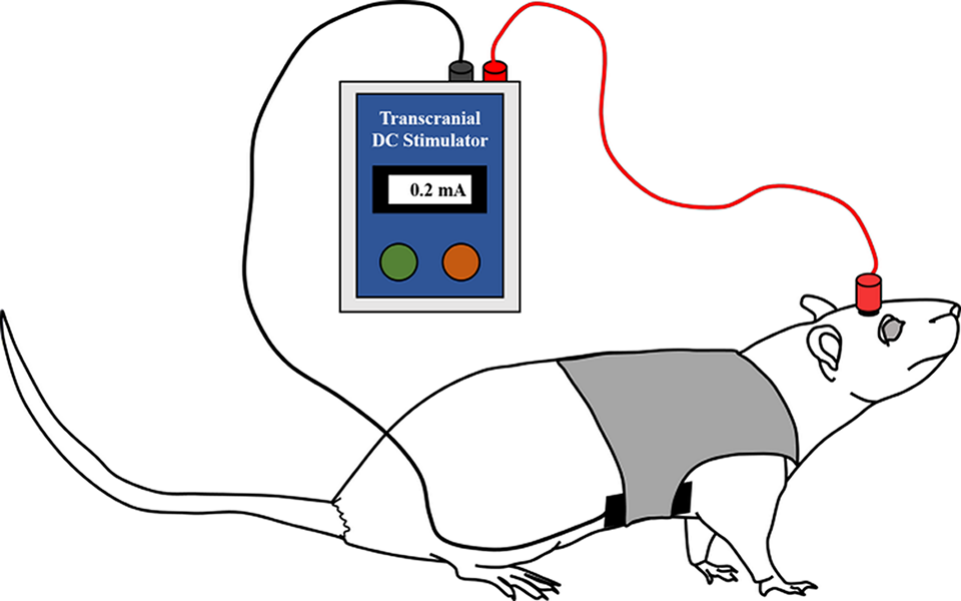
Illustration of the anodal tDCS stimulation in the rats. tDCS (0.2 mA for 20 min) or sham stimulation was applied to the rats in each group immediately after surgery. The anodal (active) electrode was placed over the right frontal area, and the cathodal (reference) electrode was placed on the ventral torso. The active electrode was filled with conductive gel, and the reference electrode was fixed using a saline-soaked sponge pad and corset.

An animal tDCS stimulator was used to produce a constant current (Soterix Medical; New York, NY, USA). **Figure 3** displays a simple schematic of the stimulation process. In the tDCS and sham groups, tDCS or sham stimulation was applied for 20 min immediately after the electrode implant surgery using the already placed epicranial electrode holder in the right frontal area (outer diameter of active electrode: 2 mm). The right frontal area was set as an active stimulation region using the anode, and a reference electrode as the cathode was placed under the ventral torso using the saline-soaked (0.9% NaCl) sponge pad (3 × 5 cm) and corset. The active electrode was filled with conductive gel to ensure maximal electrical conductance.

To improve cognitive function, including spatial learning and memory, Yu et al. applied anodal tDCS, whose intensity varies from 0.02 to 0.2 mA, and effective results occurred when the intensity was greater than 0.1 mA (40). Hence, we applied tDCS with an intensity of 0.2 mA continuously for the tDCS group. Rats in the sham group also experienced the same discomfort due to electrode placement and the sponge pad and corset for 20 min. However, no current was delivered to these rats. All rats were allowed to move freely during the stimulation.

### Behavioral Tests

**Figure 4:**
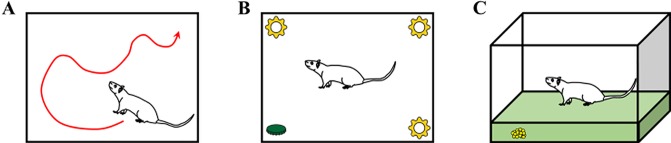
Descriptions of the three behavioral tests: **(A)** open field test, **(B)** novel object recognition test, and **(C)** buried food test. Rats in the control group (without surgery and stimulation), sham group (with surgery and sham stimulation), and tDCS group (with surgery and stimulation) underwent three different behavioral tests. The tests were performed at all time points (24 h before surgery and 6, 9, 24, and 48 h after surgery). Some of the rats (n = 10, 6 from the control group and 4 from the surgery group with depth electrodes) underwent behavioral testing at an additional time point (1 week after surgery).

All rats performed three behavioral tests sequentially in the following order as shown in **Figure 4**: open field test, novel object recognition test, and buried food test. The tests were performed 24 h before the surgery (baseline) and 6, 9, 24, and 48 h after the surgery. Although rats in the control group did not undergo surgery, the behavioral tests were performed at the same time points to enable comparisons with rats in the surgery groups (tDCS and sham groups). Behavioral assessments were also performed in 10 rats (6 from the control group and 4 from the surgery group with depth electrodes) 1 week after the surgery to monitor the recovery of the rats when compared to baseline.

The open field test was performed to measure the general activity of the rats. Prior to the baseline measurement, each rat was placed into the open field chamber for 10 min per day for three consecutive days for familiarization. During the test, the rats were placed at the center of an open field chamber (70 × 70 × 50 cm) and were allowed to move freely for 5 min. The total distance moved (in cm) was measured using Ethowatcher (Borland Software Corporation; Scotts Valley, CA, USA) (41) software, and the results were manually reviewed. Next, the mean speeds of the rats were calculated. After the test, the floor of the area was cleaned using 70% ethanol solution.

The novel object recognition test was performed to assess the attention levels of the rats in an open field chamber (50 × 30 × 40 cm). In this test, four objects were placed in the chamber, each near a corner of the chamber floor. Before the baseline measurement, each rat was familiarized with the objects placed in the four corners of the chamber for 10 min per day over 3 days. After the familiarization step, the novel object recognition test was performed at each time point. During the test, the rats were exposed to three familiar (old) objects and one new object placed at the corners of the arena. The new object exploration time was measured in relation to the time spent exploring all objects (old and new).

The buried food test was also conducted to evaluate attention at a more intuitive level (42, 43). To perform the buried food test, we provided each rat with several food pellets before the surgery for at least 3 days. This is because some rats may tend to avoid the pellets when they are offered for the first time. The rats were also habituated for 10 min per day over 3 days by placing them in the testing cage. After strict diet restriction (only 10% of food was provided when compared to the usual diet) for 1 week, several small pieces of pellet were buried in a spot 0.5 cm below the surface of clean bedding. The pellets were therefore not visible. The locations of the pellets were randomly changed each time we performed the test. The rats were placed in the center of the test cage, and the time required to eat the pellet was measured. The time to eat the pellet was defined as the time at which the rat was placed in the cage until the time at which the rat found the pellet and grasped it with forepaws or teeth. The rats were observed for a maximum 5 min, even if they could not find the food. If the rat failed to eat the pellet, it was assigned a time of 300 s. The cage was always cleaned with 70% ethanol solution after each test.

### Local Field Potential Recording and Data Acquisition

After the implantation of microelectrodes, LFPs were recorded from the rat brain 6, 9, 24, and 48 h after the surgery. The electrodes were connected to recording devices (g.USBamp and g.HEADstage, g.tec medical engineering GmbH; Graz, Austria), which acquired LFP data at a sampling rate of 1,200 Hz in the freely moving rats for 15 min at the target brain areas. After the three behavioral tests, LFPs were continuously recorded in freely moving rats for 15 min in the target brain areas.

### Data Preprocessing and Analysis

The recorded LFP data were band-pass filtered at a frequency range of 1–60 Hz. After band-pass filtering, the data were fragmented into consecutive 2-s epochs without overlapping. Using visual inspection methods, contaminated parts of the data were manually discarded. Due to only 220 epochs surviving from the noisiest data among the data set, all data sets were fitted to 220 epochs length through random selection. We then down-sampled the data from 1,200 to 100 Hz, and calculated the relative power for each channel, time (6, 9, 24, and 48 h after surgery), and frequency (gamma: 25–50 Hz, beta: 12–25 Hz, alpha: 8–12 Hz, theta: 4–8 Hz, delta: 1–4 Hz, window length: 2 s). Functional and effective connectivity among the channels were investigated at each time and frequency point using phase locking values (PLVs) and spectral Granger causality (spectral GC), respectively. PLV can measure the phase synchronization of two signals, which are simultaneously recorded from different parts of the brain. The degree of synchronization represents the neural integration between brain regions (44, 45). The directionality of neural interactions in the brain can be assessed by spectral GC. From these causalities, information flow of the brain was investigated (e.g., the concept of bottom-up and top-down processing) (46, 47).

To calculate relative power, Welch’s method was applied to each epoch of LFP data. Then, power spectral density (PSD) at the entire frequency range (1–60 Hz) can be estimated. The PSD was divided into each frequency range (gamma, beta, alpha, theta, and delta), and the PSD of each frequency range was normalized by the power of the entire frequency range.

In the case of connectivity analysis, we used PLV (functional connectivity) and spectral GC (effective connectivity). To obtain PLV at each frequency range (gamma, beta, alpha, theta, and delta), phase information of LFP data was extracted using a Hilbert transform. After averaging the differences of phase between two signals from different brain regions, the functional connectivity between brain regions can be estimated (44, 45). By means of multivariate autoregressive modeling, spectral GC estimated the causality between two signals from different brain regions. The model order was automatically decided using Akaike information criterion. Finally, the causality was calculated with respect to each frequency range (gamma, beta, alpha, theta, and delta) (46, 47).

### Statistical Analysis

The Brunner and Langer method (48) was utilized to find the main effect time (five time points: baseline and 6, 9, 24, and 48 h after surgery), group (three groups: tDCS, sham, and control group), and the interaction effect of group and time. These analyses were also performed in the same way using only two groups: surgery group and control group. *Post hoc* Mann–Whitney test was utilized to investigate differences in behavioral measures between two groups (e.g., surgery group versus control group, or tDCS group versus sham group), or those between two time points within groups (e.g., baseline versus at 6 h after surgery). As the Mann–Whitney test uses nonparametric methods, median and interquartile ratio were reported accordingly. These nonparametric tests were also utilized for neurophysiology data, except for the data from the depth electrodes. In the neurophysiology analyses using only the data from depth electrodes, independent t-tests were applied due to the very limited sample size, which can cause major confusion in the process of ranking. P-values less than 0.05 were considered statistically significant. Statistical analyses were performed using SPSS 18 (IBM Corporation; Armonk, NY, USA) and R version 3.5.3. Considering the possible confounding effect of brain destruction due to the additional insertion of depth electrodes to the thalamus, we excluded the animals with depth electrodes (n = 4) in all EEG analyses focusing only on the cortex level, and all behavioral comparisons between tDCS group and control group.

The correlations among behavioral performance in the buried food test and the EEG results were examined by only using the animals with electrodes implantation (cortex level: n = 16, thalamus level: n = 4). We only focused on the neurophysiology results exhibiting statistically significant differences between the tDCS and sham groups. All of the correlation analyses were performed using Spearman correlation analysis.

## Results

### Behavioral Measurements Exhibit an Acute Attentional Deficit and its Fluctuation

Firstly, no significant side effects were found following tDCS stimulation (e.g., Seizure) up until the end of the behavioral tests. In the analysis using three groups (tDCS, sham, and control groups), the Brunner and Langer method revealed significant main effects of group [F(2, 9) = 5.87, *p* = 0.004] and time [F(4, 9) = 5.69, *p* = 0.001] in the buried food test. In the open field test, significant main effects of group [F(2, 9) = 7.44, *p* < 0.001], time [F(4, 9) = 12.16, *p* < 0.001], and interaction effect [F(8, 81) = 2.94, *p* = 0.012] were found. Significant main effects of group [F(2, 9) = 6.64, *p* = 0.002] and time [F(4, 9) = 4.77, *p* = 0.003] were also found in the novel object recognition test. In the analysis of the rats divided into the surgery group and the control group, significant main effects of group [F(1, 19) = 10.30, *p* < 0.001] and time [F(4, 19) = 11.56, *p* < 0.001] were found in the buried food test. In the open field test, significant main effects of group [F(1, 19) = 17.73, *p* < 0.001], time [F(4, 19) = 22.87, *p* < 0.001], and interaction effect [F(4, 361) = 3.07, *p* = 0.028] were found. Significant main effects of group [F(1, 19) = 14.34, *p* < 0.001], time [F(4, 19) = 14.50, *p* < 0.001], and interaction effect [F(4, 361) = 4.72, *p* = 0.002] were also found in the novel object recognition test. As shown in **Table 1**, the rats in the surgery group had fluctuations in behavior in the buried food test after the surgery compared to baseline. In contrast, rats in the control group did not exhibit significant fluctuations in behavior at any time point when compared to baseline. In the open field test, only rats in the surgery group exhibited decreased movement when compared to baseline. This may have been due to discomfort and pain after the anesthesia and surgery. In the novel object recognition test, a similar pattern was found in both the surgery and control groups. Particularly, the exploration time shows an overall decreasing pattern over time in all groups. We were unable to find significant differences in the behaviors of the 10 rats (6 from the control group and 4 from the surgery group with depth electrodes) tested 1 week after the surgery when compared to baseline in any task. The results of individual rats for all behavioral experiments are presented in **Table S1**.

**Table 1:** The effects of the anesthesia and surgery on behavior in rats when compared to baseline. In both groups, behavioral test data were compared to baseline data at each time point in order to evaluate the effects of anesthesia and surgery. ↑ and ↓ indicate significant increases and decreases, respectively. In the buried food test, significant fluctuation was only found in the surgery group. Only the surgery groups displayed decreases in mean speed in the open field test at all time points. This suggests that there was impairment of behavior in the rats. The significance level was defined as *p* < 0.05 in the Mann–Whitney test.

Surgery group (n = 20)	After 6 h	After 9 h	After 24 h	After 48 h
Buried food test (time to find a pellet)	**—** (*p* = 0.242)	**↑** (*p* = 0.012)	**—** (*p* = 0.289)	**↑** (*p* = 0.045)
Open field test (mean speed)	**↓** (*p* = 0.005)	**↓** (*p* < 0.001)	**↓** (*p* = 0.002)	**↓** (*p* < 0.001)
Novel object recognition test (novel object exploration)	**—** (*p* = 0.091)	**—** (*p* = 0.088)	**↓** (*p* = 0.038)	**↓** (*p* = 0.019)
Control group (n = 6)	After 6 h	After 9 h	After 24 h	After 48 h
Buried food test (time to find a pellet)	**—** (*p* = 0.818)	**—** (*p* = 0.240)	**—** (*p* = 0.937)	**—** (*p* = 0.818)
Open field test (mean speed)	**—** (*p* = 0.394)	**—** (*p* = 0.310)	**—** (*p* = 0.132)	**—** (*p* = 0.818)
Novel object recognition test (novel object exploration)	**—** (*p* = 0.699)	**—** (*p* = 0.937)	**↓** (*p* = 0.009)	**↓** (*p* = 0.009)

### Comparison of Behavior Between the Transcranial Direct Current Stimulation and Sham Group

**Figure 5:**
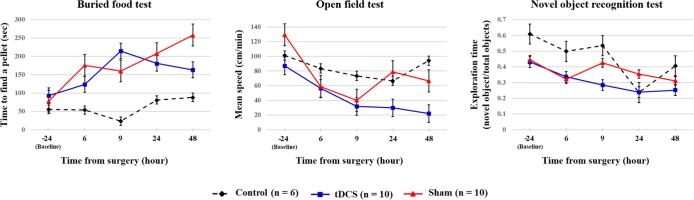
Summary of the behavioral test results of each group. The black dotted, blue, and red lines indicate control group, tDCS group, and sham group, respectively. The values are mean and standard errors.

Possible differences between the surgery groups (tDCS and sham groups) and the control group were investigated. It was found that rats in the surgery group required significantly longer time to find the food 9–48 h after surgery. Rats in the surgery group also exhibited decreased movement 48 h after the surgery. However, any significant difference was not observed in the novel object recognition test (**Table 2**, **Figure 5**). We then compared the tDCS and sham groups, as indicated in **Table 3**. In the buried food test, rats in the sham group required longer to find the pellet than those in the tDCS group 48 h after surgery. In addition, rats in the tDCS group exhibited significantly decreased movement speed than those in the sham group 24 and 48 h after the surgery. In the novel object recognition test, there were no differences between the tDCS and sham groups.

**Table 2:** Comparison of behavioral test results between the surgery and control groups. The surgery group (tDCS and sham groups) and control group were compared using three behavioral tests at each time point. Nine to 48 h after surgery, the time required to find the pellets was significantly increased in the surgery group. Moreover, 48 h after surgery, the rats in the surgery group displayed significantly reduced movement. The significance level was defined as *p* < 0.05 in the Mann–Whitney test.

	Surgery group (n = 20)		Control group (n = 6)		*p* value
	Median	Interquartile range	Median	Interquartile range	
**Buried food test** (time to find pellets, seconds)					
Baseline	58.00	106.50	42.00	72.00	0.273
After 6 h	85.00	259.00	42.00	27.00	0.138
After 9 h	252.00	240.50	12.00	18.50	0.001**
After 24 h	300.00	218.25	30.00	76.00	0.027*
After 48 h	300.00	204.25	49.00	84.50	0.027*
**Open field test** (mean speed, cm/min)					
Baseline	97.50	87.27	112.09	42.96	1.000
After 6 h	58.02	53.03	88.17	49.54	0.201
After 9 h	19.70	14.96	69.20	67.24	0.088
After 24 h	33.20	50.68	68.36	34.92	0.260
After 48 h	34.82	60.62	77.61	81.32	0.039*
**Novel object recognition test** (novel object exploration time/total exploration time, ratio)					
Baseline	0.48	0.25	0.62	0.08	0.069
After 6 h	0.28	0.34	0.53	0.12	0.233
After 9 h	0.23	0.40	0.62	0.22	0.484
After 24 h	0.28	0.40	0.24	0.33	0.572
After 48 h	0.18	0.52	0.47	0.04	0.213

tDCS, transcranial direct current stimulation.

*<0.05, **<0.005.

**Table 3:** Comparison of behavioral test results between the tDCS and sham groups. To determine the effects of tDCS stimulation, the tDCS and sham groups were compared using three behavioral tests. Twenty-four to 48 h after surgery, the rats in the tDCS group exhibited significantly decreased movement. Rats in the sham group required more time than those in the tDCS group to find the pellets 48 h after surgery. The significance level was defined as *p* < 0.05 in the Mann–Whitney test. Considering the possible confounding effect of brain destruction due to the additional insertion of depth electrodes to the thalamus, we excluded the animals with depth electrodes (n = 4) in this comparison.

	tDCS group (n = 8)		Sham group (n = 8)		*p* value
	Median	**Interquartile range**	Median	**Interquartile range**	
**Buried food test** (time to find pellets, second)					
Baseline	51.50	57.25	55.50	119.25	0.451
After 6 h	56.00	66.75	300.00	259.00	0.059
After 9 h	300.00	119.00	210.50	240.50	0.706
After 24 h	300.00	199.50	300.00	198.00	0.794
After 48 h	194.50	250.75	300.00	0.00	0.046*
**Open field test** (mean speed, cm/min)					
Baseline	96.46	32.94	119.98	114.53	0.462
After 6 h	60.23	61.46	48.95	43.17	0.834
After 9 h	18.39	12.55	18.21	12.71	0.600
After 24 h	18.51	19.28	41.81	40.55	0.036*
After 48 h	9.77	8.83	52.11	41.83	0.020*
**Novel object recognition test** (novel object exploration time/total exploration time, ratio)					
Baseline	0.40	0.26	0.43	0.27	0.958
After 6 h	0.30	0.47	0.23	0.24	0.526
After 9 h	0.20	0.18	0.52	0.83	0.631
After 24 h	0.00	0.52	0.35	0.24	0.166
After 48 h	0.00	0.45	0.19	0.53	0.721

*<0.05.

### Potential Effect of Transcranial Direct Current Stimulation on Electrophysiological Changes Due to the Onset of Delirium

**Figure 6:**
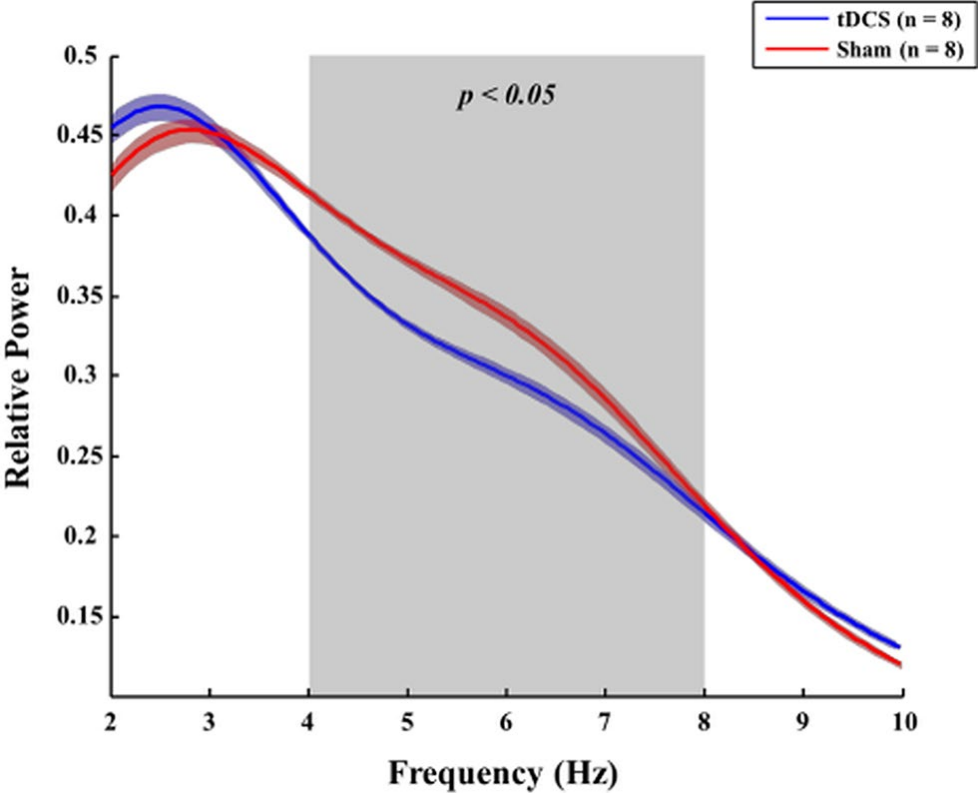
Comparison of the relative power between the tDCS and sham groups. Forty-eight hours after surgery, rats in the tDCS group (blue line) exhibited lower relative theta power in the right frontal lobe than those in the sham group (red line) (*p* = 0.023). The bold line indicates the mean value, and the shaded bands show the standard error. Mann–Whitney test was utilized.

**Figure 7:**
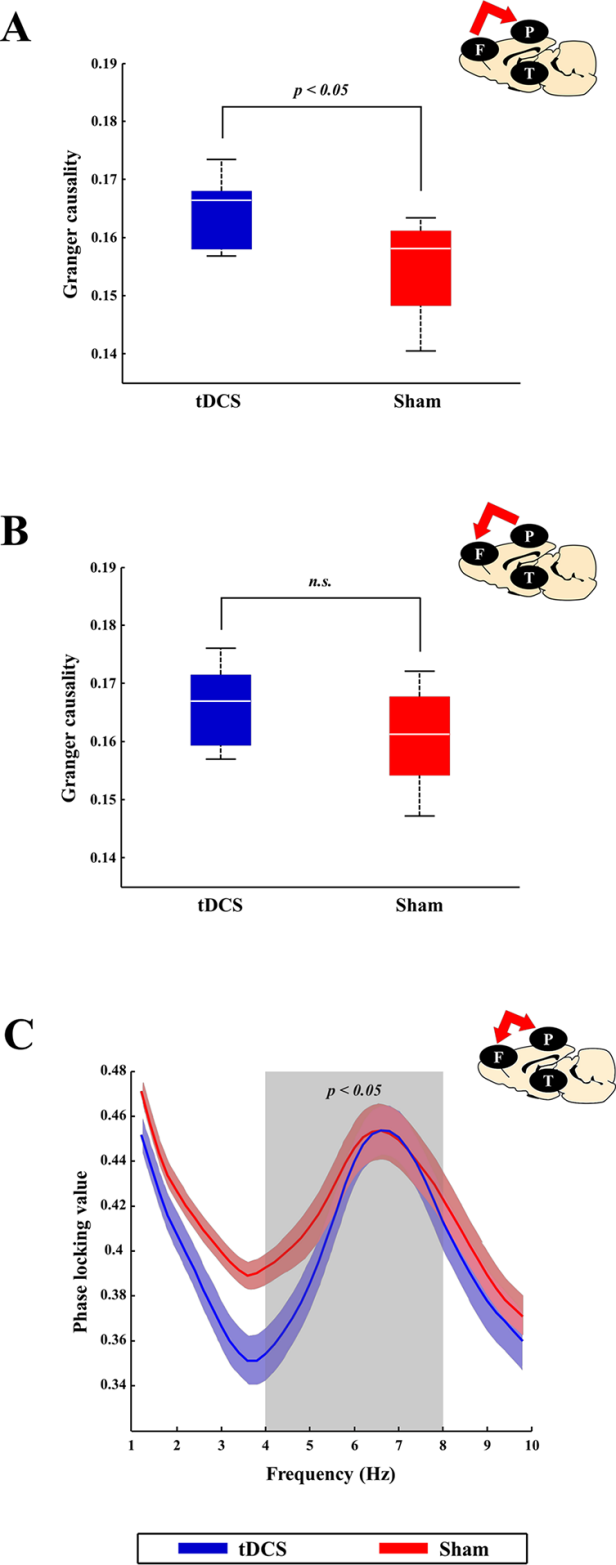
Comparison of connectivity between the tDCS and sham groups at the cortex level. At the cortex level, brain connectivity in the tDCS group tended to increase bottom-up attention (which is known to be related to gamma frequency band), which, in turn, led to recovery of awareness. tDCS stimulation helped increase bottom-up attention by enhancing gamma band effective connectivity between the frontal and parietal lobes. **(A** and **B)** The blue (tDCS group) and red (sham group) boxes represent the spectral GC values between the frontal and parietal lobes 24 h after surgery in the gamma frequency band. Effective gamma band connectivity between the frontal and parietal lobes seemed to be higher in the tDCS group than in the sham group, although only the causal relationship from the frontal cortex to the parietal cortex has a statistically significant difference (*p* = 0.047). **(C)** The blue (tDCS group) and red (sham group) lines indicated the phase locking values (PLVs) between the frontal and parietal lobes 24 h after surgery in the theta frequency band (which is known to be related to top-down attention). Rats in the sham group displayed more synchronization than those in the tDCS group (*p* = 0.016). The bold line indicates the mean value, and shaded bands show the standard error. Mann–Whitney test was utilized in all analyses.

When the Brunner and Langer method were applied on the EEG data obtained from the right frontal lobe, significant main effects of group were found only in the theta band [F(1, 7) = 13.10, *p* < 0.001]. In terms of main effects of time or interaction effect, no significant effects were found. In the *post hoc* analyses, rats in the tDCS group exhibited decreased relative theta power in the right frontal lobe when compared to those in the sham group 48 h after surgery (*p* = 0.023), as shown in **Figure 6**. In terms of other frequency bands, no significant differences were found at 48 h after surgery in the right frontal lobe (gamma: *p* = 0.945, beta: *p* = 0.547, alpha: *p* = 0.383, and delta: *p* = 1.000). The spectral GC analysis (**Figure 7**) revealed that rats in the tDCS group had more effective connectivity from the frontal to the parietal lobes in the gamma band than those in the sham group 24 h after the surgery. In particular, there was a statistically significant difference between the tDCS group and the sham group with respect to the connectivity from frontal lobe to parietal lobe (*p* = 0.047; **Figure 7A**), but not vice versa (*p* = 0.078); however, a trend was seen (**Figure 7B**). The PLV data showed that rats in the tDCS group had decreased theta synchronization between the frontal and parietal lobes when compared to those in the sham group 24 h after surgery (*p* = 0.016), as indicated by the PLV (**Figure 7C**). When assessing the relationship between the cortex and thalamus, it was found that rats in the tDCS group had more delta synchronization than those in the sham group between the parietal lobe and thalamus 24 h after the surgery (*p* = 0.015; **Figure S1C1**). In contrast, the tDCS group exhibited less gamma synchronization between these two areas than the rats in the sham group 24 and 48 h after the surgery (*p* = 0.048 and *p* = 0.018, respectively; **Figures S1A1** and **S1B1**). It was also found that effective connectivity in the gamma band from the thalamus to the parietal lobe was lower in the tDCS group when compared to that in the sham group 24 h after the surgery (*p* = 0.013). Rats in the sham group displayed almost no phase difference in the gamma frequency band between the parietal lobe and the thalamus 48 h after the surgery (**Figure S1B2**). Twenty-four hours after the surgery, the gamma and delta frequency bands had nearly synchronized phases in the above areas in the tDCS group (**Figures S1A2** and **S1C2**). The above relationships between the different brain areas are summarized in **Figure S2**.

### Correlations Among Behavioral Performances and Electrophysiology Results

A significant negative correlation was found between the time required to find food 48 h after the surgery and delta synchronization between the parietal lobe and thalamus 24 h after the surgery (*r* = −1, *p* < 0.001). A positive correlation was also found between theta synchronization between the frontal and parietal lobes and relative theta power 48 h after the surgery in the right frontal lobe (*r* = 0.582, *p* = 0.023). No significant correlations were found among the other behavioral and neurophysiological results.

## Discussion

The goal of this study was to elucidate the possible effects of single-session tDCS on postoperative delirium including cognitive and behavioral changes in rats. This study was designed based on the possibility that the acute neurobiological changes related to postoperative delirium occurs within a short critical period during or after surgery, and with evidence that the effect of single-session tDCS could persists 1 week after stimulation (49). We first determined whether microelectrode implant surgery under anesthesia induced core symptoms of delirium in rats. We found that neurosurgery under isoflurane anesthesia induced an acute onset of decreased attention and awareness, which was resolved after 1 week. In other words, rats in the control group performed well in every behavioral test during the course of the study, while those in the tDCS and sham groups exhibited fluctuating performance, meaning that the surgery group showed decreased performance in various time points during the course of the study. This finding is in line with those of previous studies using surgery under anesthesia (19, 20), suggesting that the model used here may be appropriate as an animal model of postoperative delirium. It should be noted that previous studies also reported fluctuations in the symptoms, which is one of the important criteria of delirium. Our results demonstrate that the rats prominently displayed certain symptoms, while other symptoms were mild or absent at the same time point. In addition, rats in the surgery groups displayed temporal fluctuations of symptoms compared to baseline, especially in the buried food test, where their symptoms were clearly resolved after 1 week. However, it should be noted that patients are generally not completely recovered from delirium with cognitive sequelae in clinical situations (50) so that it is desirable to prevent delirium if possible. Surgery can lead to pain and inflammation, and there is evidence that pain and inflammation can impair attention in rats (51), and anesthesia may be associated with cognitive deficits (52). The above processes may also be related to biochemical and cellular changes, such as increased levels of α-synuclein and s100β in the cortex (19), increased amyloid-β levels and tau phosphorylation in the hippocampus (53), as well as reduced levels of adenosine triphosphate (20).

When compared to the rats in the control group, those subjected to surgery and anesthesia displayed decreased movement 48 h after surgery during the open field test. The rats subjected to surgery also displayed a significant decrease in movement postoperatively when compared to baseline. It should be noted that the rats in the sham group were more active than those in the tDCS group 24 and 48 h after the surgery. In fact, among the rats that underwent surgery, all the rats showed decreased movement after surgery compared to baseline except only three rats (one of tDCS group, two of sham group). One rat in the tDCS group showed transient hyperactivity after 6 h, one rat in the sham group showed continuous hyperactivity after surgery, and the other rat in the sham group showed excessive activity from 24 h. Considering that the prevalence of hypoactive delirium is generally higher than the hyperactive presentation, these rare hyperactive rats might be linked to hyperactive delirium. Taken together, this result seems to be largely due to the performance of the rats with hyperactive delirium. Rats in the tDCS group required less time to find the food in the buried food test than those in the sham group 48 h after the surgery, suggesting that tDCS helped the rats in the tDCS group recover from postoperative delirium earlier than those in the sham group. We found no significant difference between the tDCS and sham groups in the novel object recognition test. The overall decreasing pattern of exploration time in the test over time in all groups, including the control group, might be due to the familiarization effect; however, we treated the rats to be familiarized with the test space before surgery, making a familiarization effect less likely. Intact attention, memory, and consciousness are needed to perform in both the novel object recognition and buried food tests. However, the novel object recognition test is more focused on cognitive learning and recognition memory (54), while the buried food test is more related to the natural olfactory behavior required for survival in animals (55). Considering these things, tDCS may have a possible preventive effect on the impairment of consciousness during the postoperative period, not on the higher-level cognitive function.

Several positive effects of tDCS were found on the neurophysiological measures during postoperative delirium. First, tDCS exhibited a potential to ameliorate the increase in theta power in the right frontal lobe observed 48 h after surgery in the sham group. Low-frequency activities, including those in the theta and delta bands, have been linked to cognitive impairments including dementia (56), delirium (12), and Parkinson’s disease (57). Therefore, possible effects of tDCS on theta activity may be related to the behavioral improvements observed in the tDCS group when compared to the sham group.

The effective connectivity results indicate that tDCS may have a potential to enhance the communication from the frontal cortex to the parietal cortex in the gamma band when compared to sham stimulation. There was a greater tendency to transmit information from the parietal cortex to the frontal cortex after tDCS as well. Considering that the frontoparietal effective connectivity in the gamma band was found to be important in consciousness and bottom-up attention (58, 59), which are impaired in patients with delirium, tDCS may have positively influenced the rats in our study.

The thalamus plays a critical role in our attention process (60). Therefore, the thalamus might be closely related to the pathogenesis of delirium. In fact, the thalamus was hypothesized to be a core region of drug-induced delirium as psychotropic drugs may have a potential to influence the thalamic gating function, leading to sensory overload or hyperarousal (61). This gating system abnormality might also be normalized by tDCS, and our PLV results could partially support this hypothesis. More specifically, lower gamma band synchronization between the parietal lobe and thalamus in the tDCS group seems to be more related to the normal gating function of the thalamus to prevent the overflow of bottom-up information (which is known to be related to gamma band) to the cortex level. Similarly, we found lower gamma band effective connectivity from the thalamus to the parietal lobe in the tDCS group. However, it should be noted that our interpretation of the EEG data using the depth electrodes could not lead to firm conclusions at this point due to the limitation of a small sample (n = 4) and possible brain damage caused by the implanted electrodes. In other words, our interpretation is very speculative at this point.

Many of our connectivity results did not have direct relationships with the behavioral results. We believe that it might have been difficult to identify such associations accurately due to the maximum limit value in the buried food test as well as our small sample size. Furthermore, there was a significant correlation between different time points (e.g., EEG results at 24 h and behavior results at 48 h), which should be interpreted carefully. It was expected that theta power would increase as time to find food increased in tandem; however, this was not found. This lack of finding might be due to the small sample size as well, and might also be due to slowing being a general feature, which can be shown in rats without delirium in different states such as sleepiness.

Our study has several limitations. First, our sample size was relatively small, especially in the analysis using depth electrodes. It should be considered that this study has an exploratory nature, and this was the first study that serially and simultaneously examined both the behavior and EEG changes in an animal model of delirium, specifically at the subcortex level. Considering that one of the crucial limitations of previous studies on animal models of delirium was that they investigated only the cortex level (15–17), our results are meaningful despite the small sample size. In addition, this was the first study investigating the potential effect of tDCS on core symptoms of delirium regardless of animal or human. Thus, we expect future studies would solve the small sample size problem. Second, although attentional deficits were exhibited in the rats, our results were not sufficient to clearly show the fluctuating course. Furthermore, it cannot be assured that all the rats had symptoms of delirium because surgery and anesthesia may have different impacts according to the individual subject. However, several main effects of time were found in our results using Brunner and Langer method, which could be evidence supporting fluctuating symptoms. Nonetheless, when comparing group means, we showed the induction of delirium symptoms and the possible positive effects of tDCS. Third, it is difficult to completely exclude the possibility that the microelectrodes for EEG measurement influenced the delivery of electrical stimulation. Fourth, since we only examined the effect of tDCS on EEG compared to the sham group, and not compared to baseline EEG data, our design was unable to clearly show the modulating effect of tDCS on EEG. For example, although our results showed lower theta activity in the tDCS group compared to the sham group, previous investigations on tDCS showed mixed results regarding whether it increases or decreases low-frequency EEG activities (62–64). Finally, about 4-month-old male rats were included. Given that delirium can occur in both male and female, and it does not occur frequently in young age, our results could not be generalized. Therefore, future studies may be better to use aged rats and should include both male and female animals.

## Conclusions

In this study, we developed an animal model of delirium after neurosurgery and isoflurane anesthesia, and assessed the symptoms of delirium in this model using both electrophysiology and behavioral testing. Moreover, we found evidence that anodal tDCS over the right frontal cortex has the potential to modulate aberrant neural activity and connectivity in animal models of postoperative delirium. Overall, tDCS might have the potential to ameliorate attentional deficits in patients with delirium. The present findings provide new insight into the pathogenesis and prevention of delirium.

## Ethics Statement

This study was carried out in accordance with the recommendations of the Institutional Animal Care and Use Committee (IACUC) of the Gwangju Institute of Science and Technology (GIST). The protocol was approved by the institutional review board of GIST.

## Author Contributions

All authors contributed conception and design of the study; JO and JH performed the study; JO, JH and DC analysed the data; JO, JH and BL wrote the manuscript; JP, J-JK and BL provided comments, additions, and further improvements. All authors contributed to manuscript revision, read and approved the submitted version.

## Funding

This research was supported by a grant from the Korea Health Technology R&D Project through the Korea Health Industry Development Institute (KHIDI), funded by the Ministry of Health and Welfare, Republic of Korea (Grant No.: HI16C0132). This work was also supported by GIST Research Institute (GRI) grant funded by the GIST in 2019.

## Conflict of Interest Statement

The authors declare that the research was conducted in the absence of any commercial or financial relationships that could be construed as a potential conflict of interest.
