# Supplementary material for: The Effects of Transcranial Direct Current Stimulation on the Cognitive and Behavioral Changes After Electrode Implantation Surgery in Rats
Source: Front Psychiatry. 2019 May 7;10:291. doi: 10.3389/fpsyt.2019.00291 (PMC6531794; doi:10.3389/fpsyt.2019.00291)
Supplement: Supplementary file 1 [file DataSheet_1.docx]

Supplementary Material

# Supplementary Figures and Tables

## Supplementary Figures


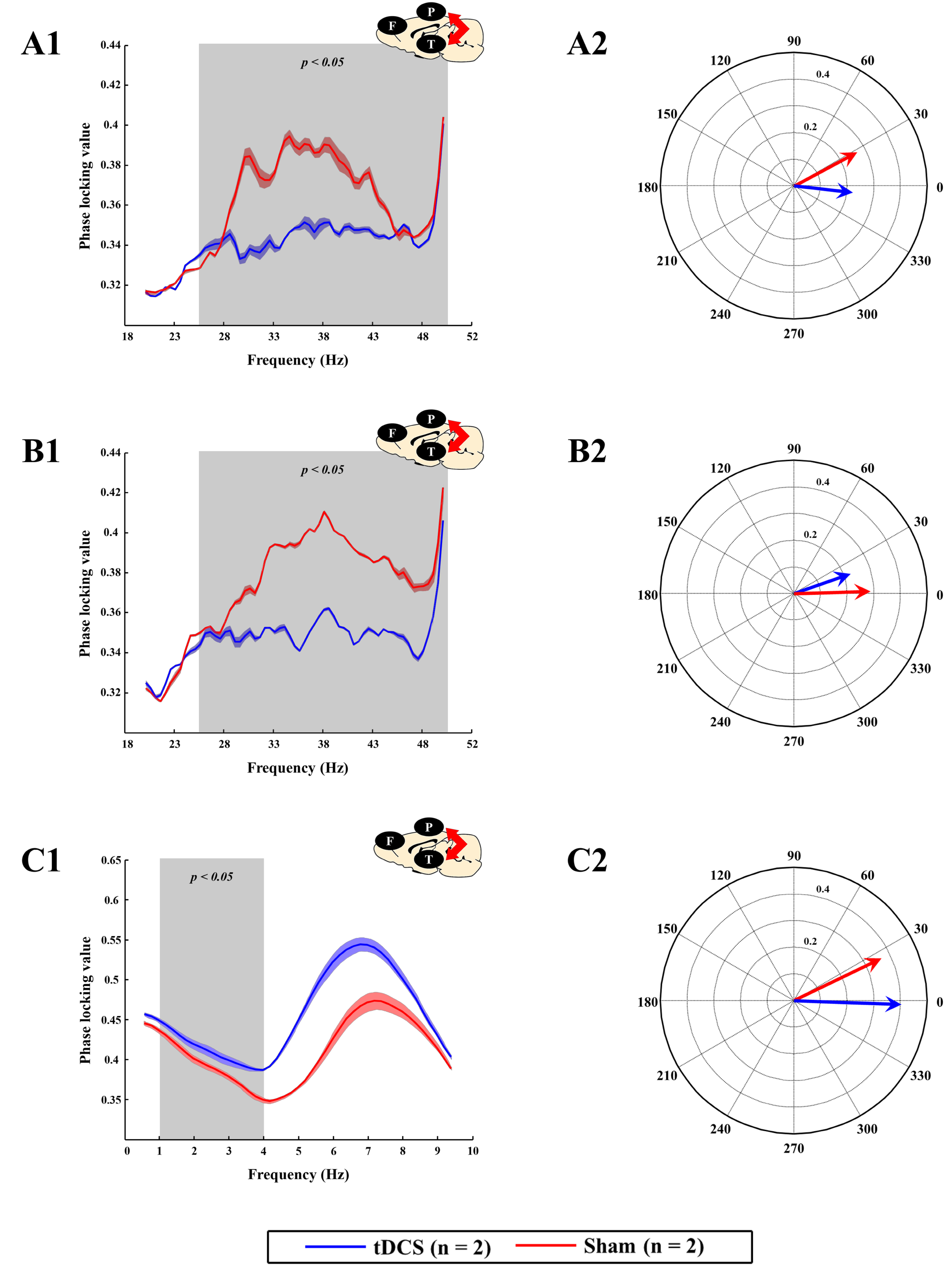


**Supplementary Figure 1.** Comparison of connectivity between the tDCS and sham groups at the cortex and deep brain levels. At the cortex and deep brain levels, connectivity in the tDCS group tended to decrease bottom-up attention to prevent excessive information flow from the thalamus. The blue (tDCS group) and red (sham group) lines indicate the PLVs between the parietal lobe and thalamus (VL). In addition, the arrows with the different colors represent phase differences in the same region in each group. The bold line indicates the mean value and the shaded bands show the standard error. (A1) and (B1) The PLV between the parietal lobe and thalamus 24 and 48 hours after the surgery in the gamma frequency band. The PLV was significantly increased in the sham group when compared to the tDCS group (*p* = 0.048 and *p* = 0.018, respectively). (C1) The PLV between the parietal lobe and thalamus 24 hours after surgery in the delta frequency band. The PLV was increased in the tDCS group when compared to that in the sham group (*p* = 0.015). (A2) The phase difference between the parietal lobe and thalamus 24 hours after surgery in the gamma frequency band. In the sham group, the phase difference between the parietal lobe and thalamus was close to 30 degrees. However, in the tDCS group, the phase difference between the 2 areas was around zero. (B2) In the sham group, the phase difference between the parietal lobe and thalamus was nearly zero 48 hours after surgery in the gamma frequency band. However, almost 20 degrees of phase difference were present between the 2 areas in the tDCS group. (C2) In the tDCS group, the phase difference between the parietal lobe and thalamus was almost zero in the delta frequency band 24 hours after surgery. However, the phase difference between the 2 areas was close to 30 degrees in the sham group. Independent sample t-test was utilized in all analyses.


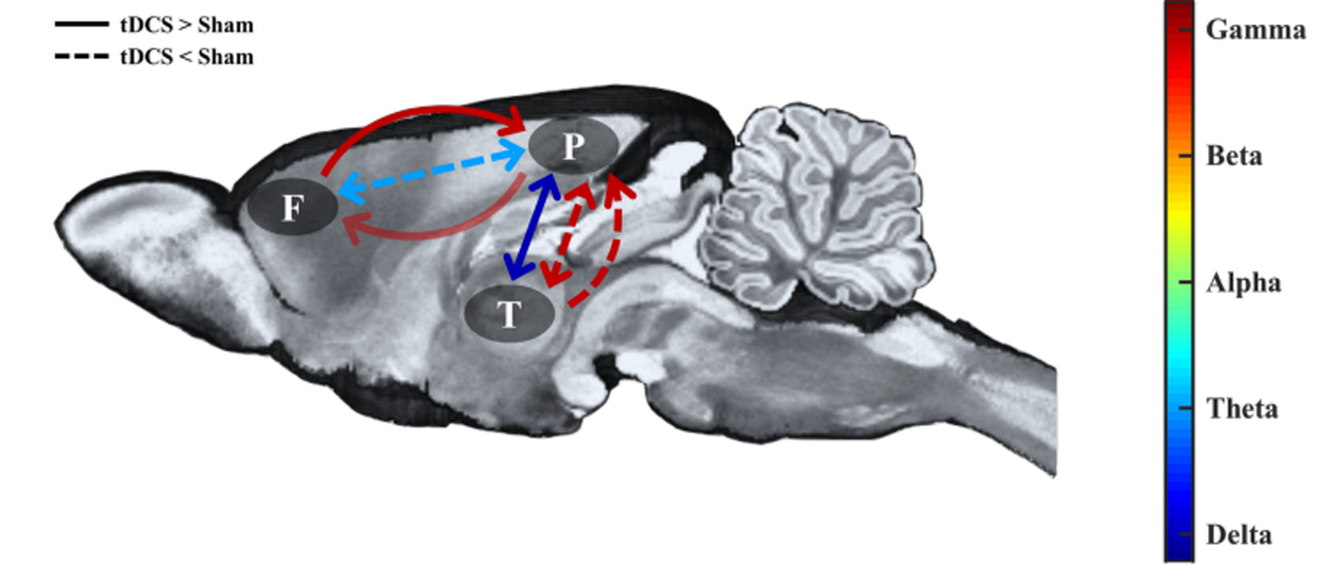


**Supplementary Figure 2.** Summary of connectivity results revealing significant differences between the tDCS and sham groups. Frontoparietal effective gamma connectivity was increased and theta synchronization between the frontal and parietal lobe was decreased in the tDCS group (after 24 hours). Delta synchronization was increased in the tDCS group between the parietal lobe and thalamus (after 24 hours), while there was less gamma synchronization exhibited between the 2 areas (after 24 and 48 hours). Gamma band effective connectivity from the thalamus to the parietal lobe was decreased in the tDCS group (after 24 hours). All connectivity results showed significant differences except for the effective connectivity from the parietal to the frontal lobe, which only showed a statistical tendency. F = frontal lobe, P = parietal lobe, and T = thalamus. Colors indicate the frequency (blue: delta, sky blue: theta, red: gamma). Unidirectional arrows indicate effective connectivity and bidirectional arrows indicate PLVs. Solid lines indicate that the connectivity values in the tDCS group were higher than those in the sham group and the dotted lines indicate the opposite.

## Supplementary Tables

**Supplementary Table 1.** Performances of individual rats in each behavioral experiment.

C = control group (n = 6), T = tDCS group (n = 10), and S = sham group (n = 10)

B = baseline, A6h = After 6 hours, A9h = After 9 hours, A24h = After 24 hours, and A48h = After 48 hours.

|  | Buried food test  (time to find pellets, seconds) | | | | | Open field test  (mean speed, cm/min) | | | | | Novel object recognition test  (novel object exploration time /total exploration time, ratio) | | | | |
| --- | --- | --- | --- | --- | --- | --- | --- | --- | --- | --- | --- | --- | --- | --- | --- |
|  | B | A6h | A9h | A24h | A48h | B | A6h | A9h | A24h | A48h | B | A6h | A9h | A24h | A48h |
| C1 | 15 | 28 | 28 | 13 | 71 | 129.63 | 160.64 | 150.5 | 106 | 160.66 | 0.51 | 0.52 | 0.42 | 0.54 | 0.47 |
| C2 | 97 | 42 | 8 | 300 | 300 | 121.1 | 37.3 | 71.1 | 7.3 | 60.73 | 0.62 | 0.47 | 0.6 | 0.28 | 0.44 |
| C3 | 129 | 14 | 6 | 10 | 109 | 103.08 | 97.38 | 113.78 | 97.56 | 165.96 | 0.62 | 0.62 | 0.7 | 0 | 0.57 |
| C4 | 63 | 131 | 5 | 47 | 10 | 78.36 | 97.13 | 67.3 | 70.3 | 64.86 | 0.5 | 0.85 | 0.71 | 0.19 | 0 |
| C5 | 21 | 64 | 76 | 103 | 27 | 38.2 | 29 | 25.4 | 52.3 | 90.35 | 0.62 | 0.53 | 0.64 | 0.41 | 0.49 |
| C6 | 6 | 42 | 16 | 13 | 11 | 136.69 | 79.2 | 12.3 | 66.42 | 22.27 | 0.78 | 0 | 0.14 | 0 | 0.47 |
| T1 | 10 | 105 | 204 | 300 | 89 | 70.63 | 132.47 | 80.44 | 46.77 | 41.58 | 0.35 | 0.27 | 0.28 | 0.53 | 0.41 |
| T2 | 55 | 82 | 300 | 300 | 41 | 155.48 | 102.58 | 30.76 | 34.75 | 32.37 | 0.57 | 0.51 | 0 | 0 | 0 |
| T3 | 300 | 300 | 300 | 300 | 300 | 25.97 | 14.5 | 12.92 | 7.3 | 7.3 | 0 | 0 | 0 | 0 | 0 |
| T4 | 48 | 62 | 300 | 300 | 300 | 82.16 | 58.01 | 13.81 | 22.35 | 10.8 | 0.83 | 0.11 | 0.36 | 0.52 | 0.56 |
| T5 | 74 | 25 | 112 | 300 | 52 | 95.71 | 69.43 | 20.83 | 27.88 | 7.75 | 0.34 | 0.32 | 0.22 | 0.73 | 0 |
| T6 | 32 | 9 | 300 | 60 | 300 | 97.21 | 16.85 | 2.25 | 14.66 | 6.77 | 0.45 | 0 | 1 | 0 | 0 |
| T7 | 132 | 50 | 300 | 114 | 300 | 157.82 | 62.44 | 24.6 | 8.89 | 11.17 | 0.58 | 0.67 | 0.16 | 0 | 0 |
| T8 | 29 | 3 | 17 | 26 | 27 | 97.79 | 11.39 | 15.95 | 10.8 | 8.74 | 0.23 | 0.96 | 0.17 | 0 | 0.98 |
| T9 (depth) | 187 | 300 | 300 | 89 | 38 | 80 | 54.6 | 98.4 | 96.6 | 78.1 | 0.53 | 0.29 | 0.43 | 0.44 | 0.38 |
| T10 (depth) | 62 | 300 | 13 | 17 | 185 | 7.3 | 41.6 | 16.2 | 28.1 | 15.4 | 0.43 | 0.23 | 0.22 | 0.17 | 0.19 |
| S1 | 50 | 45 | 63 | 104 | 300 | 63.3 | 18.67 | 18.56 | 117.81 | 92.13 | 0.51 | 0 | 1 | 0.45 | 0 |
| S2 | 149 | 300 | 300 | 300 | 300 | 114.35 | 84.25 | 17.85 | 31.65 | 84.77 | 0.37 | 0.20 | 0.89 | 0.59 | 0 |
| S3 | 26 | 300 | 300 | 300 | 300 | 125.6 | 39.87 | 11.07 | 42.8 | 64.52 | 0 | 0.21 | 0 | 0.41 | 0.73 |
| S4 | 29 | 300 | 300 | 300 | 300 | 55.49 | 23.65 | 9 | 15.89 | 9.38 | 0.61 | 0.35 | 0 | 0.74 | 0 |
| S5 | 174 | 300 | 300 | 300 | 300 | 240.19 | 63.26 | 23.3 | 61.2 | 39.7 | 0.50 | 0.50 | 0.80 | 0.28 | 0.59 |
| S6 | 30 | 300 | 49 | 300 | - | 202.58 | 7.7 | 5 | 19.27 | - | 0.27 | 0 | 0 | 0.09 | - |
| S7 | 61 | 27 | 41 | 96 | - | 166.98 | 58.03 | 23.25 | 92.83 | - | 0.61 | 0.51 | 0.81 | 0.28 | - |
| S8 | 149 | 29 | 121 | 37 | 116 | 29.27 | 72.5 | 27.81 | 40.81 | 37.27 | 0.25 | 0.26 | 0.24 | 0.14 | 0.37 |
| S9 (depth) | 29 | 88 | 34 | 300 | 149 | 127.8 | 91.7 | 189.21 | 242.08 | 132.7 | 0.81 | 0.62 | 0.30 | 0.29 | 0.62 |
| S10 (depth) | 82 | 66 | 92 | 38 | 300 | 169.18 | 125.8 | 73.78 | 124.62 | 72.3 | 0.52 | 0.56 | 0.21 | 0.27 | 0.18 |
